# Application research of learning curve in the training of pharmacists for the review of narcotic and psychotropic drug prescriptions

**DOI:** 10.3389/fmed.2026.1803176

**Published:** 2026-07-16

**Authors:** Jingjing Xu, Min Xu, Xue Wang, Huijun Ren, Jiao Luo, Heng Xi, Qin He

**Affiliations:** Department of Pharmacy, The Third People’s Hospital of Chengdu, Chengdu, Sichuan, China

**Keywords:** anesthetic and psychotropic drugs, learning curve, pharmacist, pharmacist competency training, prescription review

## Abstract

**Background:**

Learning curves can quantify how trainees acquire complex clinical skills. We applied this concept to track pharmacists’ proficiency in reviewing prescriptions for anesthetics and Schedule-I psychotropics—high-risk areas where workforce shortages are acute.

**Methods:**

Twenty pharmacy trainees enrolled in a tertiary-level hospital in 2025 completed a 10-step program; each step comprised 100 prescription reviews. Accuracy of review decisions, time to completion, and correct use of professional terminology were recorded for every trainee at every step. Cumulative-sum charts for binary outcomes were constructed for each metric, for the cohort overall, and for each individual. Pearson and Spearman coefficients tested associations between the step at which a trainee’s curve flattened and baseline characteristics.

**Results:**

Group-level curves plateaued at step 7 for overall proficiency, step 6 for terminology, step 7 for accuracy, and step 8 for speed. Individual curves began to flatten between steps 4 and 9. A strong negative correlation was found between years of prior front-line pharmacy experience and the number of steps required to reach plateau (*r* = −0.521, *p* = 0.018).

**Conclusion:**

Learning-curve analysis objectively identifies when pharmacists achieve consistent, timely, and accurate review of high-risk prescriptions. The cohort approached proficiency after approximately 600–700 reviewed prescriptions, with prior experience accelerating the process. Incorporating curve monitoring into training programs can guide individualized instruction and provide an evidence-based benchmark for competence.

## Background

In China, high-quality pharmaceutical professionals in China are predominantly concentrated in large urban hospitals, while primary healthcare institutions such as county-level hospitals, township health centers, and community health service centers generally face challenges including insufficient pharmacist staffing and varying levels of professional competence. To address this, the National Health Commission (NHC) collaborated with the Ministry of Finance to formulate the 2025 Medical Service Capacity Enhancement Plan. This initiative implements standardized “theory + practice” intensive training programs in tertiary hospitals (training bases), enabling pharmacists from diverse regions to master unified and standardized prescription review and medication monitoring protocols, thereby ensuring patients receive pharmaceutical services of comparable quality across different geographical areas. This represents a pivotal strategy for China to strengthen talent foundations for modernization through systematic educational reforms, industry demand orientation, and optimized talent policies. Against this backdrop, our hospital, as a large-scale tertiary Grade A hospital and pharmacist training base, undertakes the critical and challenging task of prescription review and dispensing training (1).

Narcotic drugs and Category I psychotropic substances (collectively referred to as “NPS”) pose unique potential risks, particularly characterized by high addictive properties. Abuse not only causes severe and irreversible harm to individual health but may also lead to social issues. Consequently, the state has classified them as specially regulated pharmaceuticals under stringent monitoring, establishing rigorous standards for dispensing and prescription review. Pharmacists must possess strong professional competence when reviewing such prescriptions, making enhanced training in NPS prescription review capabilities particularly essential to ensure medication safety.

To address the current lack of quantitative assessment tools in prescription review training for pharmacists at primary care hospitals, this study introduces the learning curve as an analytical framework. The learning curve graphically represents the relationship between accumulated practice (*X*-axis) and performance (*Y*-axis), typically characterized by an initial slow phase, a rapid improvement phase, and a plateau. In health professions education, learning curves can be statistically modeled using parameters such as baseline level, learning rate (slope), and maximal performance (upper asymptote) (2). In prescription review training, behavioral data such as review time and error rates can be tracked over practice sessions, and pharmacists’ competency stages can be inferred based on curve characteristics (e.g., slope and plateau phases) (1, 3, 4). The learning curve CUSUM method (5, 6), which enables quantitative monitoring of individual skill acquisition, has been applied to procedural training in emergency medicine (5) and offers a scientific basis for evaluating pharmacist training outcomes.

As one of the national-level pharmacist training bases, the Pharmacy Department of our hospital innovatively applied the learning curve theory teaching model in the prescription review training for young pharmacists from primary hospitals during the training program for psychotropic and narcotic drugs. Through comprehensive and meticulous evaluation of trainees’ learning processes and data collection, the CUSUM (cumulative sum) method—a statistical technique that detects systematic changes in sequential performance data by cumulatively summing deviations from a target value—was employed to quantitatively identify the point at which prescription review competency reached a stable plateau, thereby enabling real-time tracking of growth and scientific assessment of training effectiveness. Based on these findings, training strategies were optimized to enhance efficiency and reduce resource consumption. This study provides scientific support for training model optimization, not only improving the quality of training at our hospital but also offering new insights for optimizing training models in other positions.

## Methods

### Participants

In this study, 20 trainees who were enrolled in the prescription review position training for urgently needed pharmacists at Chengdu Third People’s Hospital in 2025 were included. Inclusion criteria were: ① Possession of a valid pharmacist qualification certificate; ② Pharmacists engaged in prescription review work in primary healthcare institutions; ③ Voluntary participation in the study with signed informed consent forms. Exclusion criteria included: ① Planned consecutive absences exceeding 2 weeks during the study period (impossibility of completing full training or assessments); ② Concurrent participation in other similar training programs or studies that may cause interference.

### Design

The department has established a teaching and evaluation team, consisting of four teachers with intermediate or higher professional titles, extensive experience in managing narcotic and psychotropic drugs, and teaching abilities. In terms of teaching methods, the team strictly follows the teaching syllabus to provide systematic prescription review training and assessment for students. The training content is rich and targeted. Firstly, a total of 4 h of theoretical learning are arranged, which comprehensively covers various knowledge points and practical skills related to prescription review of narcotic and psychotropic drugs. After the theoretical teaching is completed, students enter the practical assessment stage. During this stage, the instructors accurately evaluate the progress of students’ practical skills according to the data collection requirements of the learning curve. At the same time, a specially-assigned person is responsible for collecting data throughout the entire practical process, tracking it until each student successfully completes the required number of prescription reviews. After each round of prescription review practice, the instructors aggregated the identified problems and provided detailed feedback and explanations to the trainees, enabling them to correct errors and improve their performance in subsequent rounds.

To ensure consistency and reliability in the scoring of student practical assessments by different instructors, we have established a unified and standardized standard process (7), as follows: (1) Strengthening the professional training of scorers: Organize prescription review scorers to participate in systematic and professional training courses. Through comprehensive and in-depth training, we aim to help scorers accurately grasp the key points of evaluation, enhance the precision and consistency of the evaluation, and ensure that each scorer can conduct the review work with unified and accurate standards. (2) Clarifying the detailed rules for prescription review: Develop detailed and clear prescription review specifications, clearly defining the review content, review standards, review process, and the weight allocation of each item. This provides scorers with clear guidelines and a basis for reference during the review process, ensuring the standardization and rigor of the review work from a systemic perspective. (3) Implementing a dual review guarantee mechanism: Introduce a dual review system, where each prescription is reviewed by one scorer and then re-checked by another, minimizing the impact of individual subjective factors on the review results and further enhancing the reliability and credibility of the evaluation results. (4) Building a scientific system for quantitative scoring: Adopt scientific and reasonable quantitative evaluation methods, using carefully formulated detailed scoring rules and pre-set reasonable weights to quantitatively score prescription reviews. Based on objective data and clear indicators, we ensure that the evaluation process is not influenced by subjective judgments, effectively reducing deviations and making the evaluation results more objective and accurate (8). Specific evaluation indicators and operational requirements for prescription review ability are detailed in Table 1.

**Table 1:** Evaluation indicators and operational requirements for practical ability in prescription review of anesthetic and psychotropic drugs during.

Quantized value	Learning project	Operational requirements and standards	Target value
A1	The accuracy rate of review result determination	Prescription review involves the following three aspects: (1) Legality review. Check whether the prescription signature and seal are from a person with prescription authority; review whether the signature style of the prescribing physician is consistent with the sample retained by the pharmacy department to prevent signature forgery or omission. (2) Normative review: The prescription modification is not standardized, lacking signature and date; the prescription is incomplete and not fully written (3) Suitability review: ① Consistency between prescribed medication and clinical diagnosis; ② Inconsistency between prescribed dosage and trial dosage; ③ Inappropriate frequency of administration; ④ Inappropriate route of administration; ⑤ Prescribed dosage exceeding legal limit; ⑥ Prescribed dosage exceeding maximum limit; ⑦ Inappropriate selection of medication; ⑧ No indication for clinical diagnosis; ⑨ Duplicate medication; After all 20 individuals have completed the prescription review task for this stage, the accuracy rate for each individual and the overall compliance rate will be calculated; every 100 prescriptions constitute one stage.	100% correct (Failure rate 0.1)
A2	review speed	The pharmacist receives prescriptions pending review, judges their reasonableness, and points out any issues with those that raise objections. Every 100 prescriptions constitute one stage, and the overall timing is recorded.	≤60 min/100 sheets (Failure rate 0.1)
A3	Normativity of prescription reviewer’s expression	After completing the staged prescription review task, conduct simulated practice exercises for trainees’ system operation abilities. Each trainee reviews 100 prescriptions in each stage and is able to express the problems in a professional manner. They are graded on five dimensions, including: accurately identifying the type of problem (2 points); accurately describing the prescription problem (2 points); and pointing out the correct prescribing method (2 points), for a total of 6 points.	≧5 points (Failure rate 0.05)

The target value is set based on the average daily work level of pharmacists dispensing anesthetic and psychotropic drugs. Since no standard target value for this skill has been found in the literature, this study integrates various aspects such as laws and regulations, professional norms, medical quality, experience feedback, and industry trends, and combines the actual situation of the position to set three reasonable and practical prescription review assessment indicators to improve review accuracy and efficiency (9). The acceptable failure rate for each target value is determined based on historical data and industry standards: (1) accuracy rate of review results (A1): National regulations mandate 100% accuracy for narcotic prescriptions, but single-person review cannot achieve this consistently in daily practice, necessitating a second check. The target is 100% per 100 prescriptions with a 10% failure rate, derived from a six-month analysis of trainee error logs in our hospital, which showed an initial error rate of 8–12%. The target value is set at 100% accuracy per 100 prescriptions, with a failure rate of 10%; (2) prescription review duration (A2): according to timed measurements of experienced pharmacists indicated an average of 60 min per 100 prescriptions, based on pilot data where 90% of initial blocks were completed within 66 min, the target time is 60 min per 100 prescriptions, with a failure rate of 10%; (3) prescription expression standardization (A3): Routine evaluations require a score ≥5 out of 7 for system proficiency. The 5% failure rate (stricter than A1/A2) reflects that documentation is more trainable and less variable; this threshold was established by the teaching team from 3 years of internal records according to daily work, proficiency in system use is required, with a score of 5 or above. The target value is set at 5 points, with a failure rate of 5%. The quantified value of the study subjects’ prescription review ability is ∑ = A1 + A2 + A3.

### Observation indicators

The question bank contains 1,000 prescriptions, which are randomly divided into 10 groups using a random number generator, with each group consisting of 100 prescriptions as a learning stage, and there are a total of 10 learning phases, thus forming a complete practical operation learning process. This grouping method ensures that each prescription has an equal probability of being assigned to any group, guaranteeing the randomness of the grouping, including the randomization of the difficulty, problem type, and correct/incorrect ratio of prescriptions in each group. This avoids learning differences that may arise due to prescription selection bias, helping us to observe more objectively and accurately the changes in students during the learning process, laying the foundation for scientifically evaluating learning outcomes. During the practice process, after the prescription review task for each stage is completed, teachers will promptly provide guidance and corrections to students based on the problems found during the review, helping them continuously improve their prescription review abilities. At the same time, we collect data on the following indicators: ① Using data analysis software, we record in detail the accuracy rate determined by the review results. ② Using countdown and reminder tools, we collect the time taken for each person to review prescriptions in each stage, to derive the prescription review speed; ③ Arranging designated teachers to conduct on-site scoring, focusing on improving pharmacists’ professional competence based on professional expression methods for prescription review issues. After the review by 20 students in each stage is completed, we calculate the indicator data and failure rate for each pharmacist in each stage.

Data collection was conducted on three indicators at each stage of training, and the ability changes of pharmacists during the learning process were reflected by fitting the learning curve equation.

### Learning curve fitting

This study constructs learning curves at three levels: ① learning curves for practical skills across different indicators; ② overall learning curves for practical skills; ③ individual learning curves for prescription review capabilities among trainees.

First, descriptive analysis was conducted by creating a scatter plot. The horizontal axis (*X*-axis) represents the practice phase, while the vertical axis (*Y*-axis) shows the average values of all samples across specific indicators within each phase, providing an intuitive visualization of the overall progress trend of trainees in various indicators.

Subsequently, the cumulative sum analysis (CUSUM) method for binary variables was applied (1, 10). Based on samples with a sample size *n* > 1, the CUSUM statistics for each indicator were calculated. The quantified value *A* for prescription review capability was derived using the formula: *A* = *Xj* − *X*0, where *Xj* reflects whether each indicator met the target value at each practice stage (i.e., the probability that trainees failed to achieve the target value after completing each stage), and *X*0 represents the target failure rate. The CUSUM statistic was computed as: $\mathrm{Sj}=\sum_{j=1}^{i}(\mathrm{Xj}-X0)$ representing cumulative summation. This CUSUM statistic quantifies deviations between actual performance and target values across stages through cumulative summation.

Construct a functional relationship curve with the practice stage as the *X*-axis and the CUSUM statistic as the *Y*-axis. Perform functional fitting on the curve (selecting the optimal model based on the coefficient of determination *R*^2^) to calculate the slope *k*. When *k* ≤ 0, the corresponding x-coordinate value represents the peak point of the curve. When *k* ≤ 0, the corresponding horizontal coordinate (*X*-axis) value represents the peak point of the curve. The number of practice stages (*Y*-axis) corresponding to this peak point indicates the stage at which the research subject has mastered the skill, and also reflects the minimum number of practice sessions required to acquire the skill.

Finally, the goodness of fit of the fitted curve is evaluated using the *R*^2^ value. A higher *R*^2^ value indicates a smaller degree of dispersion between the fitted curve and the original CUSUM values, suggesting better fitting quality. Higher *R*^2^ values signify that the derived functional equation possesses greater reliability, enabling more accurate representation of pharmacists’ learning trajectories and competency enhancement pathways during prescription review processes.

### Statistical analysis

Data analysis was conducted using SPSS 23.0 statistical software. Categorical data were analyzed using the Chi-square test, polynomial regression, and segmented model fitting curves. Correlation analysis was performed using Pearson (continuous variables) or Spearman (categorical variables), and a multiple linear regression model was established for further analysis.

## Results

In the practical arrangement, after random grouping of prescriptions for each stage of the hemp extract treatment, the correct ratio of prescriptions, as well as the proportion of problematic prescriptions in terms of legality, standardization, and appropriateness, were statistically analyzed. No significant differences were observed in the difficulty of prescription review across different stages. Details are provided in Table 2.

**Table 2:** Comparison of Prescriptions for Anesthetic and Psychotropic Drugs at Different Learning Stages.

Stage	Number of prescriptions	Correct prescription	Problem prescription		
			Legality issues	Standardization issues	Appropriateness issues
Stage 1	100	22	18	25	35
Stage 2	100	32	16	18	34
Stage 3	100	25	20	21	34
Stage 4	100	23	15	19	43
Stage 5	100	24	19	17	40
Stage 6	100	19	21	26	34
Stage 7	100	21	17	24	38
Stage 8	100	30	16	18	36
Stage 9	100	23	18	19	40
Stage 10	100	25	17	19	39
*χ*^2^ value	14.566				
*p* value	0.975				

*p* < 0.05 indicates statistically significant.

### Comparison of results across different indicators

**Figure 1:**
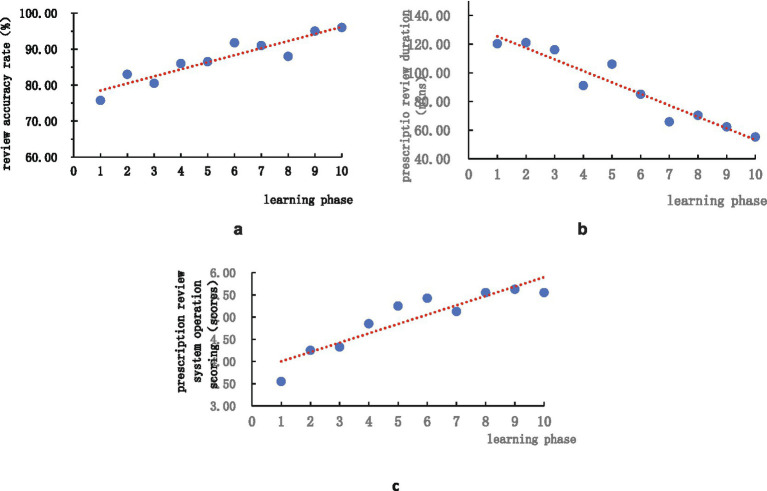
**(a)** Scatter plot trend chart of the accuracy rate of review result judgment; **(b)**: Scatter Plot Trend of Audit Speed; **(c)**: Trend chart of prescription expression standardization scores upon review.

The accuracy rate of prescription review results for 20 trainees at each stage was calculated as (88 ± 0.06) % (70–100 prescriptions). As the stage changes, the accuracy rate of prescription review results shows an upward trend (Figure 1). The prescription review duration was (89.31 ± 25.35) mins (50–130 min), and it gradually decreases as the stage changes (Figure 1b). The prescription review system operation scoring results were (4.95 ± 0.70) points (3–5 points), indicating an upward trend (Figure 1c).

### Analysis of CUSUM learning curves for different indicators

**Figure 2:**
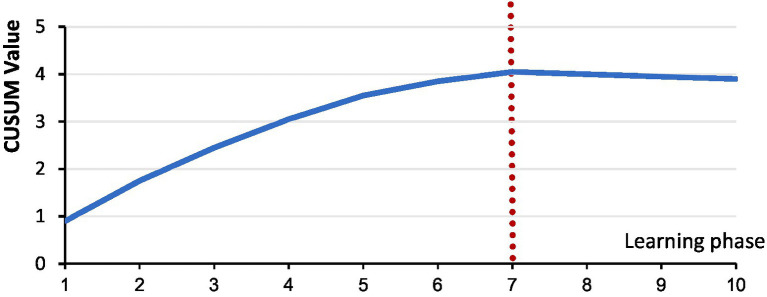
Learning curve for improving the accuracy of prescription review results for anesthetic and psychotropic drugs.

In the constructed coordinate system, the operational stages corresponding to different indicator learning curves are set as the *X*-axis, and the cumulative sum values (i.e., CUSUM values) of all research subjects at each operational stage are set as the *Y*-axis, to plot and obtain the learning curve values. The function formula for the learning curve fitted to the change in accuracy of the prescription review results by trainees is as follows: *y* = 0008524*x*^4^–0.01411*x*^3^ + 0.004526*x*^2^ + 0.8117*x* + 0.9135. The coefficient of determination *R*^2^ = 0.9994, indicating a good curve fitting effect. Taking the derivative of *y*, we obtain the slope function *k* = *y*′ = 0.0034096*x*^3^–0.04233*x*^2^ + 0.009052*x* + 0.8117. Calculate the slope k for different stages of the curve. When the value of k transitions from positive to negative, it signifies a successful leap over the learning curve. It is observed that the k value is negative in the 7th stage. From this, we can infer that after reviewing the 700th prescription, the accuracy of prescription review results has pass the peak of the learning curve, reaching the proficiency stage. See Figure 2.

**Figure 3:**
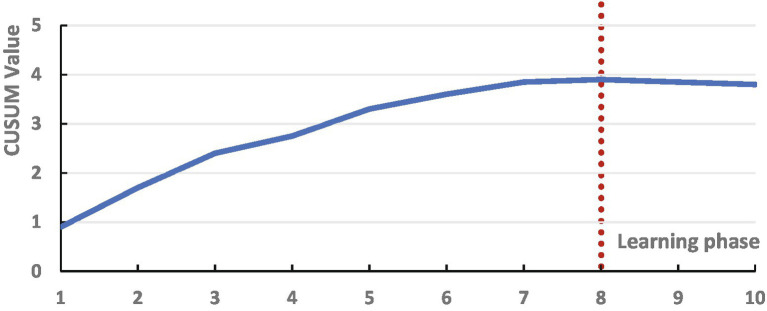
Learning curve for improving the speed of narcotic drug prescription review.

The formula for the curve fitting function of the learning curve for prescription review speed of the research subject is: *y* = −0.000987*x*^4^ + 0.02145*x*^3^–0.1762*x*^2^ + 1.038*x* + 0.9128, with *R*^2^ = 0.9992, indicating a good curve fit. Taking the derivative of y, we obtain the slope function *k* = *y*′ = −0.003948*x*^3^ + 0.06435*x*^2^–0.3524*x* + 1.038, and calculating the slope k at different stages, it is found that the *k*-value of the curve is negative at the 8th stage. When the trainee accumulatively reviews the 800th prescription during practice, their prescription review speed successfully pass the peak of surpasses the learning curve, the target and breakthrough in speed have been achieved as shown in Figure 3.

**Figure 4:**
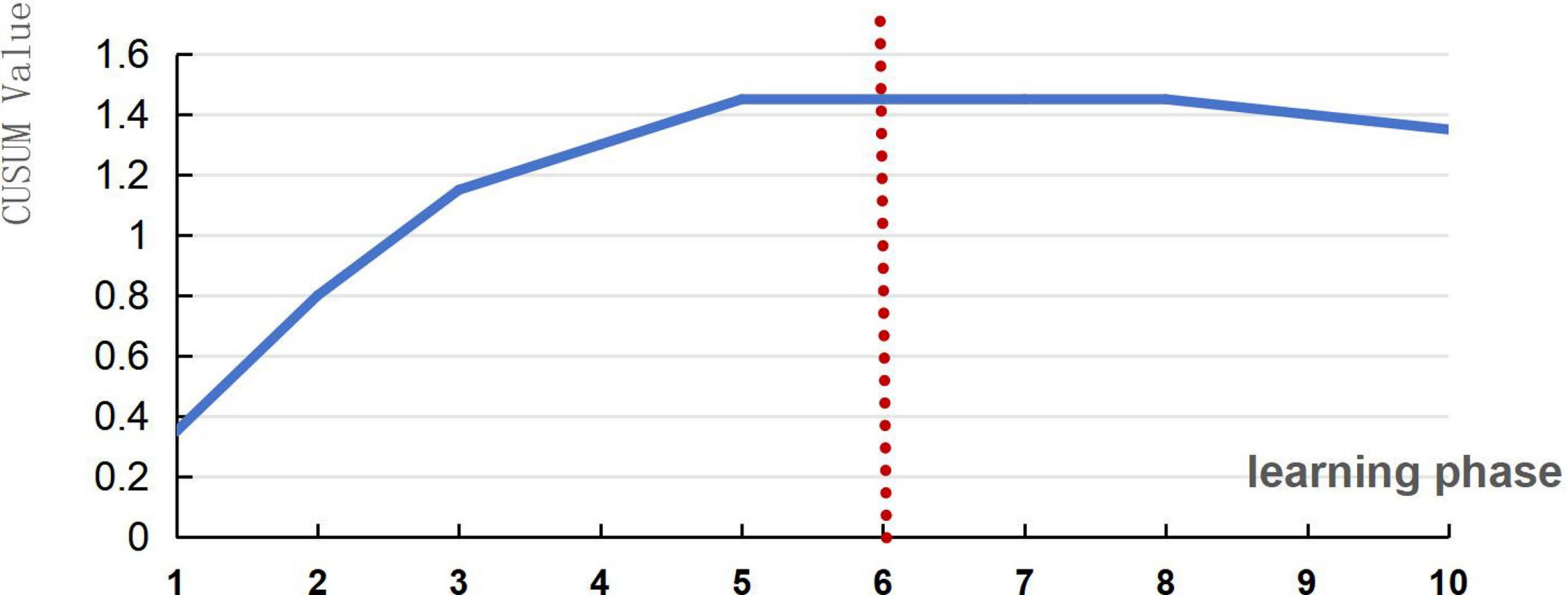
Learning curve of students’ ability to evaluate prescription expression normatively.

The learning curve fitting function formula for improving the normative ability of prescription review expression is: *y* = −0.0002039*x*^4^ + 0.006954*x*^3^–0.09523*x*^2^ + 0.5536*x* + 0.3473, with *R*^2^ = 0.9980 indicating a good curve fit. Taking the derivative of y, we obtain the slope function *k* = *y*′ = 0.0008156*x*^3^ + 0.020862*x*^2^–0.19046*x* + 0.5536, by analyzing the slope *k* of the curve in each stage, it is found that the k value of the curve is negative in the 6th stage. Therefore, after practicing until the 600th prescription, the learner’s normative ability of prescription review expression can overcome the learning curve. See Figure 4.

### Analysis results of overall practical competency learning curve

**Figure 5:**
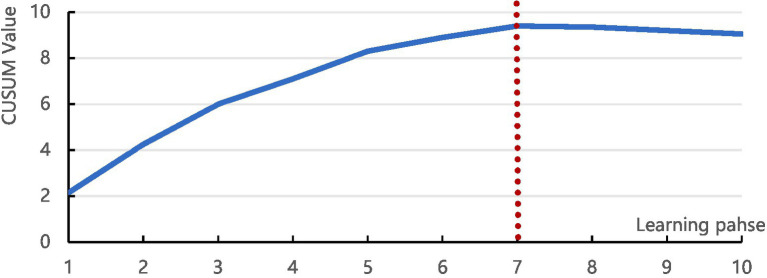
Learning curve of overall practical competence in prescription review.

The quantified value of prescription review ability for each research subject is the sum of three assessment indicators, which also represents the overall learning curve, i.e., ∑ = A1 + A2 + A3. The *X*-axis represents the operational phase of the overall learning curve, while the *Y*-axis represents the cumulative sum of each operation performed by all trainees. The CUSUM curve is plotted in Figure 5. After analysis and processing, the best-fit equation is *y* = −0.001158*x*^4^ + 0.02310*x*^3^–0.1865*x*^2^ + 1.284*x* + 2.167, with a determination coefficient *R*^2^ = 0.9987, indicating a good curve fitting effect. See Figure 5 for details.

Taking the derivative of the fitted function y, we get *k* = *y*′ = −0.004632*x*^3^ + 0.06930*x*^2^–0.3730*x* + 1.284. Calculate the derivative value of the fitted function corresponding to the k value in each stage. After the 7th stage, the CUSUM learning curve k value becomes negative, indicating that the peak point is reached during the 7th stage of prescription review. From this point, the curve turns from upward to downward. Therefore, after practicing until the 700th prescription, the trainee’s prescription review ability has crossed the learning curve.

The corresponding derivative (*k*) values for each learning phase are presented in Table 3, which quantitatively characterizes the transition from rapid learning to plateau and post-peak periods, trainees showed rapid improvement in the early stages (stages 0–3), followed by a plateau phase (stages 4–7), and a slight decline in performance after stage 7.

**Table 3:** Learning curve k-value.

Learning phase	0–1	1–2	2–3	3–4	4–5	5–6	6–7	7–8	8–9	9–10
Derivative (K)	1.284	0.976	0.724	0.0520	0.356	0.225	0.121	−0.036	−0.037	−0.102
Description of learning efficiency	Rapid learning phase			Learning plateau			Peak period	Pass the peak		

### Individual learning curve of trainee prescription review ability

By collecting basic information from 20 trainees and individualized data during the learning process of anesthetic and psychotropic drugs prescription review. The phase after the learning curve slope k decreases for each learner. It signifies that the learner has passed the peak of the learning curve, entering the phase of mastering prescription review skills. The phase at which each learner’s pass the peak of the learning curve, details are provided in Table 4.

**Table 4:** learners’ basic information and the phase of passing the peak the learning curve.

Student ID	Gender	Age	Academic qualification	Professional title	Working years in grassroots hospitals	The phase of passing the peak of the learning curve
1	Female	25	Undergraduate	Intermediate pharmacist	3	8
2	Female	35	Junior college	Intermediate pharmacist	13	8
3	Female	30	Junior college	Junior pharmacist	10	8
4	Female	31	Undergraduate	Junior pharmacist	8	7
5	Female	31	Undergraduate	Junior pharmacist	8	6
6	Male	30	Master	Intermediate pharmacist	5	5
7	Female	26	Undergraduate	Junior pharmacist	4	9
8	Female	29	Undergraduate	Intermediate pharmacist	6	8
9	Female	25	Undergraduate	Junior pharmacist	3	8
10	Female	36	Master	Junior pharmacist	10	9
11	Male	39	Undergraduate	Junior pharmacist	17	5
12	Female	35	Master	Intermediate pharmacist	9	8
13	Female	40	Master	Intermediate pharmacist	13	7
14	Female	39	Undergraduate	Junior pharmacist	17	6
15	Female	28	Undergraduate	Junior pharmacist	6	7
16	Male	29	Junior college	Junior pharmacist	9	6
17	Female	27	Junior college	Intermediate pharmacist	7	5
18	Male	30	Undergraduate	Junior pharmacist	8	5
19	Female	31	Junior college	Junior pharmacist	10	7
20	Female	34	Undergraduate	Intermediate pharmacist	11	5

All participants had worked exclusively in primary healthcare institutions since graduation; therefore, their working years in grassroots hospitals are identical to their total working years.

### Correlation between individual learning curve and basic information of learners

To further investigate the impact of learners’ basic information on the changes in their individual learning curves, we analyzed the correlation between trainees’ ability to pass the peak of the learning curves and potential influencing factors. By employing Pearson and Spearman analysis methods, we found a significant negative correlation between the time taken to pass the peak of the learning curves and working years (*r* = −0.521, *p* = 0.018). This suggests that the longer the grassroots work experience, the earlier at the stage pass the peak of the learning curves, leading to an earlier entry into the proficiency stage. Furthermore, age and work experience exhibit a high degree of collinearity. Education background, and professional title showed no significant correlation with the time taken to pass the peak of the learning curves (*p* > 0.05). See Tables 5 and 6.

**Table 5:** Linear correlation among continuous variables.

Variable	*r*	*p*
Learning phase and age	−0.412	0.072
Learning phase and working years	−0.521	0.018
Age and working years	0.912	<0.001

*p* < 0.05 indicates statistical significance, and *r* > 0.7 or <−0.7 indicates a high degree of correlation or high degree of negative correlation.

**Table 6:** Correlation between learners’ ability to pass the peak of learning curves and potential influencing factors.

Possible influencing factors	*r*	*p*
Academic qualification	0.048	0.84
Professional title	−0.293	0.208
Working years in grassroots hospitals	−0.507	0.022
Age	−0.398	0.084

*p* < 0.05 indicates statistical significance, and *r* > 0.7 or <−0.7 indicates a high degree of correlation or high degree of negative correlation.

Based on the analysis results of correlation, a multiple linear regression model was established to further explore the combined effects of various factors on the stage of pass the peak of the learning curve. Given that other variables were not significant, the model was simplified (Table 7).

**Table 7:** Analysis of the impact of age and work experience on passing the peak of the learning curve.

Variable	Coefficient	*t*	*p*
Constant term	9.177	18.18	<0.001
Years of work experience	−0.16	−2.76	0.013^*^

*p* < 0.01 indicates statistical significance.

Due to the high collinearity between the age and work experience of the trainees (Pearson *r* = 0.912), including both in the model would lead to unstable coefficients. All variables except age, as work experience has a stronger correlation with learning progress. Therefore, the only significant predictor variable is work experience in grassroots hospitals. The regression equation is: Stage of passing the peak of the learning curve = 9.177–0.160^*^ work experience. That is, for every additional year of work experience, the slope of the learning curve decreases by about 0.16 stages earlier. Work experience alone explains 27.1% of the variation in passing the peak of the learning curve (*R*^2^ = 0.271), indicating that there are still other unobserved factors affecting the timing of the emergence of passing the peak of the learning curve.

## Discussion

For the training of talents needed by the country, our hospital, as one of the training bases for pharmacist positions, has taken on the important task of prescription review and dispensing training. This work involves multiple professional requirements and complex practical operations, presenting certain difficulties and challenges (11, 12) To assess the teaching effectiveness more scientifically and accurately, this study introduces the learning curve theory into this teaching process. Although the trainees participating in this training are all from grassroots medical institutions and have some work experience, they are not familiar with the management and review of prescriptions for narcotics and psychotropic drugs in large-scale top-tier hospitals. In view of this, we have adopted a standardized training model, aiming to significantly assess trainees’ progress in mastering the prescription review work for narcotics and psychotropic drugs during the implementation process, thereby improving teaching quality and efficiency (13, 14).

According to literature reports (15–17), Miller’s Pyramid and the Dreyfus Model serve as classic competency development theories in the field of medical education., providing essential qualitative frameworks for understanding the progression of clinical skills from novice to expert levels. Miller’s Pyramid delineates competency dimensions through four stages: Knows, Knows How, Shows How, and Does. The Dreyfus Model, from a cognitive development perspective, maps learners’ progression from a rule-dependent novice stage through a situational recognition expert stage to an intuitive decision-making mastery stage. However, while these models effectively characterize competency development phases, they struggle to capture the continuous process and efficiency of skill enhancement—specifically, the “speed of improvement” and the “number of practice sessions” required for competency advancement.

The value of the learning curve theory lies in its ability to conduct quantitative analysis of this efficiency improvement process. This curve does not replace classical theories such as the Miller Pyramid and the Dreyfus Model but rather serves as a quantitative supplement to them: classical theories specify “what should be observed,” with the help of this theory, both instructors and trainees can more clearly evaluate the trajectory of skill mastery. In the initial stage, pharmacists often face numerous challenges: they may struggle to accurately determine the correctness of prescriptions, have unclear focuses, lack rigorous expression and communication, and spend a long time reviewing prescriptions (18). However, with the gradual accumulation of experience, pharmacists can gradually focus on common issues in prescription review, quickly and accurately identify errors, and propose practical modification plans. It can be seen that the learning curve theory plays a significant role in enhancing pharmacists’ prescription review abilities, which is embodied in the following aspects: (1) It establishes a quantitative analysis framework for evaluating training effectiveness. This framework helps to comprehensively and objectively assess the actual performance of pharmacists during standardized training, providing a strong basis for improving training quality. (2) By continuously tracking the specific performance of trainees at different learning stages, it is possible to quantify the speed and efficiency of their skill improvement. This allows for a more precise evaluation of the rationality of training plans, enabling optimization and adjustment of training schemes. (3) It helps to accurately identify bottlenecks and difficulties encountered by pharmacists during the learning process. After clarifying these key issues, targeted training in related areas can be strengthened, assisting pharmacists in overcoming difficulties and achieving rapid improvement in their abilities. (4) It enables a more objective and comprehensive evaluation of whether practical abilities meet established standards, effectively avoiding potential interference from luck and other factors in traditional one-time assessment models, ensuring the accuracy and reliability of evaluation results.

Upon conducting in-depth research on the learning curves of different indicators, it was found that the inflection points for the k values of the three indicators occur in the 7th, 8th, and 6th stages, respectively. Based on the feedback from trainees, the difficulties faced at the inflection point for the indicator of correct rate of audit results judgment include: (1) Being unfamiliar with the unique prescription pattern of our hospital’s Anesthesia and Precision Medicine Department. Due to a lack of exposure and understanding, trainees need a gradual process to familiarize themselves with this unique prescription pattern, which to some extent affects their ability to accurately determine the correct rate of audit results. (2) Shortcomings in professional knowledge reserves. Especially in terms of drug selection suitability, trainees’ knowledge is not solid enough, making it difficult to accurately judge whether the drug selection is suitable for the patient’s condition and physical state, thereby affecting the accuracy of audit results. (3) Insufficient understanding of relevant standards, laws, regulations, and rules for prescription audit. This makes it difficult for trainees to accurately judge whether the prescription is legal and standardized when auditing prescriptions (19). Additionally, due to a lack of practical experience, they find it challenging to make accurate judgments and handle complex situations properly, further hindering the improvement of the correct rate of audit results. In terms of improving the speed of prescription audit, trainees reach the standard relatively late. The main difficulty lies in being overly cautious during the assessment process, fearing the omission of important information. They deliberate repeatedly during the audit, making it difficult to quickly provide a clear audit result, thus resulting in a relatively slow improvement in the speed of prescription audit. As for the improvement of trainees’ ability to express prescriptions normatively, in the initial stage, trainees often find it difficult to accurately point out the problems in prescriptions using more professional terminology. This inaccuracy in expression greatly reduces communication efficiency (20, 21). However, through practice and guidance in each stage, trainees continuously accumulate experience and improve their abilities. By the 7th stage, they successfully cross the learning curve and master the ability to accurately express prescription problems using professional terminology, achieving significant improvement in the normativity of prescription expression.

Studying the overall learning curve can intuitively reflect the progress of trainees in prescription review ability (22). When the overall study subjects enter the seventh stage of practice, the k value of the learning curve shows a downward trend, and the curve shape gradually flattens. This change indicates that the learning process has entered a stable phase, meaning that the overall indicators have successfully crossed the learning curve and entered a relatively proficient operational stage. Based on this, we establish the seventh stage as a key dividing point. Reviewing the learning process before the dividing point, we can see from the change in slope k value that each trainee was in a rapid learning phase when reviewing the first to 300 prescriptions. During this period, the trainees’ familiarity with prescription review work rapidly increased, and their knowledge and skills accumulated quickly. However, when the number of reviewed prescriptions was in the training range of 301 to 700, the trainees gradually entered a slowdown and plateau phase of learning. Although the learning speed slowed down, with the continuous increase in the number of prescription reviews, the trainees made significant progress in multiple aspects: the accuracy of review results significantly improved, the review speed gradually accelerated, and professional expression ability steadily enhanced. After approximately 700 prescriptions were practiced, the trainees were able to proficiently master prescription review skills. This research result provides a reference for future training on prescription review for Schedule I drugs, a strong basis for instructors to develop teaching strategies, and also indicates the goals and directions for improving overall teaching quality.

To contextualize this finding within the broader literature, we compared it with existing studies using similar methodologies. Our finding that trainees required approximately 700 prescriptions to reach stable proficiency aligns with Xu et al. (1), who reported competency attainment between stages 6–8 using the same CUSUM framework, suggesting that mastering narcotic and psychotropic drug prescription review requires at least 600–800 practice prescriptions—far more than the 100–200 used in traditional assessments. Cross-domain comparisons with LC-CUSUM studies on endotracheal intubation (5) and anesthesiology procedures (23) indicate that cognitive tasks with high safety stakes demand as much deliberate practice as technical procedures. Thus, the late competence attainment observed here is not anomalous but highlights a general need for more practice opportunities. Moreover, learning curve analysis quantitatively supplements qualitative frameworks like Miller’s Pyramid (2) by providing empirical benchmarks—e.g., ~700 prescriptions to move from “Shows How” to “Does”—offering a more complete competency assessment.

Through a study on the learning curve of prescription review ability among 20 trainees, it was found that there were differences in the progress of proficiency in corresponding operational skills among individuals, with the stages of crossing the learning curve ranging from 4 to 9. Additionally, a significant negative correlation was observed between the time taken to cross the learning curve and age (*r* = −0.521, *p* = 0.018), suggesting that longer grassroots work experience, may be associated with an earlier decrease in the learning curve slope (the learning plateau appears earlier), and there is a high degree of collinearity between age and work experience. Gender, education, and professional title showed no significant correlation with the time taken to cross the learning curve (*p* > 0.05). Possible reasons for this, Possible reasons for this, although speculative given the small sample size, include: (1) improved learning efficiency due to accumulated experience (24). Long-term grassroots practice has enabled trainees to accumulate rich practical case experience, enabling them to identify common problem patterns in prescription review more quickly, thereby reducing the time required for basic understanding of new knowledge. (2) systematic thinking and familiarity with workflow. Long-term work has led trainees to have an internalized understanding of medical processes and standardized operations, enabling them to grasp the overall framework and key links more quickly when learning new skills, reducing adaptation time. (3) strong stress resistance and adaptability. Grassroots work experience often comes with strong environmental adaptability and task stress resistance, leading to lower anxiety and a more stable mindset when facing new skill learning, which helps to quickly enter the groove early. (4) clear problem awareness and demand orientation. Trainees with long grassroots work experience are more likely to align their learning content with practical problems, and learning oriented towards problem solving tends to be more efficient, with initial improvement being more noticeable. By establishing a multiple linear regression model to further explore the combined influence of various factors on the decline phase of the learning curve slope, the model suggested that, for every additional year of work experience, the decline phase of the learning curve slope might advances by about 0.16 stages in this sample. For example, compared to working for 3 years, working for 13 years results in the plateau phase appearing about 0.16 × 10 = 1.6 stages earlier. Furthermore, the model accounted for 27.1% of the variance in the stages of crossing the learning curve (*R*^2^ = 0.271) in this sample, suggesting that other unobserved factors may also affect the timing of the learning curve plateau phase.

The application of learning curves can provide important reference for the design of on-the-job training and teaching for pharmacists, a position in short supply. Given the late competence attainment observed in our study (approximately 700 prescriptions to reach proficiency) and its consistency with existing literature, we propose the following evidence-informed strategies to accelerate skill acquisition without compromising patient safety. These strategies should be tested in future research using the learning curve methodology: (a) Pre-training familiarization modules. A brief e-learning module introducing hospital-specific prescription formats and common errors can reduce initial skill acquisition time (25). (b) Stratified training based on baseline knowledge. Pre-training assessment to identify weaker trainees, followed by targeted remediation, can flatten the initial learning slope and reduce overall training duration (1). (c) Deliberate practice with focused feedback. Prioritizing the most frequent or highest-risk errors for immediate correction, as per Ericsson’s deliberate practice framework (24), can shorten the plateau phase. (d) Progressive responsibility design. Beginning with lower-risk prescriptions before progressing to controlled substances, following a stepwise entrustment model (26), can reduce cognitive overload and accelerate competence. The effectiveness of these strategies can be quantified by comparing learning curve parameters (e.g., slope, plateau onset) between intervention and control groups in future multi-center trials.

While these strategies offer promising directions for future research, the present study has several limitations that should be acknowledged. Firstly, no control group was designed in this study. While the teaching methods (e.g., teachers, content, objectives) were consistent with those of traditional training models, traditional assessment relies primarily on summative endpoint evaluations (e.g., final exam scores), whereas our learning curve approach focuses on process-based dynamic monitoring (e.g., review time, error rates across stages). These fundamentally different data collection methods preclude direct group comparisons between the two approaches. Future studies could address this limitation by designing a control group that receives an alternative innovative teaching method and comparing learning curve parameters (e.g., slope, plateau onset) between groups. Therefore, if future studies aim to design a control group, they can collect process data through other innovative teaching methods and compare and analyze learning curves to evaluate the impact of new methods on assessment effectiveness. Secondly, the limited number of participants in this study constrained its scope. Although a small sample size limits the generalizability of research results to some extent, this exploratory study focusing on randomly recruited participants still maintains scientific rigor and rationality (27). However, its findings only reflect the learning curve model of the participant group at this teaching base. Considering the differences in the overall level of participants recruited by different teaching bases, the following research suggestions are proposed: ① expand the sample size to include more participants with different backgrounds to enhance the representativeness and reliability of the results; ② replicate the study in different contexts to test the stability and reproducibility of the results, thereby enhancing the universality and credibility of the conclusions; ③ encourage in-depth and extensive research to verify the expanded findings, explore new perspectives, and provide comprehensive and in-depth insights for related fields. Thirdly, female pharmacists accounted for 80% (16/20) of the participants in this training program, while males constituted only 20%. This reflects the current reality of female predominance in primary healthcare pharmacy service teams in China. However, the significant gender imbalance poses challenges for statistical analysis. Due to the limited male sample size and insufficient statistical power, we did not use gender as a core grouping variable for comparative analysis. Although we excluded the main effect of gender on outcome variables, it remains uncertain whether the learning curve model derived from this study applies equally to male pharmacists. Therefore, caution should be exercised when generalizing these findings to male pharmacists. Future research should expand male sample sizes through multicenter collaboration or targeted recruitment to further examine the potential moderating role of gender in prescription review skill acquisition processes. Fourth, this study lacked systematic qualitative data collection. The learning barriers discussed (e.g., unfamiliarity with prescription patterns, knowledge gaps, overcautiousness) are based on instructor observations and informal trainee comments rather than on structured qualitative analysis. Future studies should incorporate formal qualitative methods—such as semi-structured interviews or thematic analysis of reflective journals—to better understand the learning process and validate the observed barriers. Future research should achieve multi-center coverage, incorporating students of different levels and backgrounds into the study, and introducing more innovative teaching strategies. It should observe teaching effects from a broader perspective and analyze the differences in learning curves among different students from a deeper perspective. By accurately observing students’ learning progress and precisely identifying their weak links, personalized learning and development plans can be tailored for each student. Furthermore, it is necessary to promote the collaborative advancement of teaching methods and learning modes, and build a more efficient pharmaceutical standardized training system that meets individual needs.

## Conclusion

The review of prescription for narcotic and psychotropic drugs is crucial for medical safety and rational drug use. It is a professional requirement for pharmacists to accurately master this skill, and it is also the key to ensuring the safety of patients’ medication. Therefore, the learning curve theory is extremely important for improving review ability. It can help teaching institutions design rationalized practical workload, avoid excessive repetitive teaching and resource waste; it can also accurately identify key indicators for review during the learning process, enhancing the learning effectiveness of students; at the same time, based on personalized learning situations during the assessment process, it can continuously adjust and improve teaching methods to enhance teaching quality. This enables the training of talents urgently needed by the country to be based on rigorous data and analysis, continuously optimizing teaching content and methods, and rationally allocating resources. Working together to promote the education of pharmacists’ review competency, we aim to cultivate more professional and responsible pharmaceutical talents for society, ensuring public medication safety.

## Data Availability

The raw data supporting the conclusions of this article will be made available by the authors, without undue reservation.
